# A Dynamic Interference Detection Method of Underwater Scenes Based on Deep Learning and Attention Mechanism

**DOI:** 10.3390/biomimetics9110697

**Published:** 2024-11-14

**Authors:** Shuo Shang, Jianrong Cao, Yuanchang Wang, Ming Wang, Qianchuan Zhao, Yuanyuan Song, He Gao

**Affiliations:** 1 School of Information and Electrical Engineering, Shandong Jianzhu University, Jinan 250101, China; 2 Department of Automation, Tsinghua University, Beijing 100084, China; 3 Shandong Zhengchen Technology Co., Ltd., Jinan 250101, China

**Keywords:** robot vision, underwater scene reconstruction, dynamic target detection, attention mechanism, deep learning

## Abstract

Improving the three-dimensional reconstruction of underwater scenes is a challenging and hot topic in the field of underwater robot vision system research. High dynamic interference underwater has always been one of the key issues affecting the 3D reconstruction of underwater scenes. However, due to the complex underwater environment and insufficient light, existing target detection algorithms cannot meet the requirements. This paper uses the YOLOv8 network as the basis of the algorithm and proposes an underwater dynamic target detection algorithm based on improved YOLOv8. This algorithm first improves the feature extraction layer of the YOLOv8 network, improves the convolutional network structure of Bottleneck, reduces the amount of calculation and improves detection accuracy. Secondly, it adds an improved SE attention mechanism to make the network have a better feature extraction effect; in addition, the confidence box loss function of the network is improved, and the CIoU loss function is replaced by the MPDIoU loss function, which effectively improves the model convergence speed. Experimental results show that the mAP value of the improved YOLOv8 underwater dynamic target detection algorithm proposed in this article can reach 95.1%, and it can detect underwater dynamic targets more accurately, especially small dynamic targets in complex underwater scenes.

## 1. Introduction

The development of underwater robotics technology is advancing in tandem with the increasing exploration and exploitation of oceanic resources. Vision systems are essential in underwater intelligent devices because of their non-contact measurement techniques, cost-effectiveness, and large information capacity. Attaining autonomous navigation and obstacle evasion in underwater robotics presents a considerable challenge that frequently necessitates three-dimensional reconstruction. Underwater dynamic target detection is essential for achieving 3D reconstruction [1]. The precision of underwater dynamic target recognition directly influences the quality of 3D reconstruction, subsequently affecting the efficacy of autonomous navigation and obstacle avoidance in underwater robots. Consequently, attaining high precision in dynamic target recognition is essential and significant.

The existence of dynamic disturbances in the water, including moving objects and variable environmental conditions, has resulted in considerable challenges, frequently causing blurred reconstructions, ghosting, and excessive noise. Precisely recognizing dynamic disturbances is essential for their exclusion from the 3D reconstruction process, hence enhancing the quality of the reconstruction. Underwater dynamic interference denotes visual disruptions in the aquatic environment induced by the movement of things (such as fish, submersibles, or other marine organisms) as well as natural occurrences like water currents and waves. These dynamic interferences adversely impact the quality of images taken by cameras and can induce blurriness and distortion of subjects, complicating later three-dimensional reconstruction. Precise identification of dynamic interference is crucial for its exclusion from the reconstruction process. Despite substantial progress in deep learning-based target detection networks, further improvements are necessary to provide enhanced identification results in complex and low-light underwater settings. With the progression of deep learning, research on deep learning-based object identification algorithms concentrates on improving detection efficacy, accommodating complex scenarios, and minimizing model parameters. Nonetheless, numerous challenges persist in obstructing the effective identification of items in underwater environments. These challenges encompass inadequate lighting, the similarity between targets and their environment, and the existence of substantial amounts of pollutants.

We will present a concise summary of the research undertaken on target detection methods. Object detection methodologies can be categorized into two types: one-stage and two-stage. One-stage detection methods, although faster, generally demonstrate worse accuracy than two-stage algorithms, which include a resampling operation. One-stage algorithms are often utilized in contexts where speed is prioritized over high accuracy, such as in industrial inspection and security surveillance.

In 2014, Sermanet et al. [2] introduced the OverFeat algorithm, marking the first generation of one-stage object detection algorithms. This algorithm employed a convolutional neural network (CNN) [3] to perform classification, localization, and detection tasks and introduced a multi-scale, sliding window method based on neural networks. In 2016, Redmon et al. [4] proposed the YOLO (you only look once) algorithm, which uses regression for object detection by integrating classification, localization, and detection tasks into a single network pass, directly producing bounding boxes and class probabilities from the input image. Inspired by the YOLO algorithm and the mechanism of anchor boxes, Liu et al. [5] developed the SSD (Single Shot MultiBox Detector) algorithm the same year, utilizing feature maps of different scales to detect objects of varying sizes, which enhanced detection accuracy while maintaining speed. In 2017, Redmon et al. [6] introduced YOLOv2, which improved the extraction network by removing the fully connected layers from the original YOLO and optimizing the network with Batch Normalization, thus enhancing the detection capabilities and speed. Subsequently, Redmon et al. [7] proposed YOLOv3, which incorporated ideas from Residual Networks (ResNet) [8] and upgraded the feature extraction network to Darknet-53, using three different sizes of anchor boxes for more accurate boundary box prediction and improving small object detection. In 2018, Zhang et al. [9] presented RefineDet, which adopted the idea of optimizing bounding boxes from two-stage detection algorithms and maintained detection efficiency while improving accuracy. In 2020, Law et al. [10] proposed CornerNet, which differs from previous methods by enhancing object edge detection through heatmaps and connecting vectors. Alexey et al. [11] introduced YOLOv4, utilizing CSPDarkNet-53 as the feature extraction layer and adding Spatial Pyramid Pooling (SPP) in the Neck part to expand the receptive field of the feature maps while combining Path Aggregation Network (PAN) and Feature Pyramid Networks (FPN) to enrich the detail in the fused feature maps. Shortly after, Jocher et al. [12] proposed YOLOv5, an improvement over YOLOv4, which introduced the Focus structure and used the CSP architecture to enhance layer fusion, increasing computational speed and reducing information loss. YOLOv6 [13] and YOLOv7 [14] emerged in 2022, demonstrating superior detection capabilities compared to YOLOv5. In 2023, ULTRALYTICS released YOLOv8, a significant update from YOLOv5, supporting image classification, object detection, and instance segmentation tasks with improved accuracy and throughput. The development of the YOLO series continues with the recent release of YOLOv9 [15].

Two-stage detection algorithms are also significant due to their high accuracy. A classic example is R-CNN [3], which uses a convolutional network to process images and significantly improves detection performance compared to traditional algorithms. He et al. [16] improved upon R-CNN with SPP-Net, which reduces the convolution operations to one and transfers the extraction of candidate box feature vectors to post-convolution feature maps, drastically reducing computational demand. This method introduced an SPP structure that allows feature maps of different sizes to be transformed into a uniform size, enabling the detection of images of any size. Girshick et al. [17] improved upon SPP-Net with Fast R-CNN, which introduced an ROI Pooling layer to standardize feature map sizes and enabled direct processing of entire images in the network, integrating classification loss and bounding box regression and addressing the issue of redundant candidate boxes in R-CNN. Subsequently, Ren et al. [18] introduced Faster R-CNN, significantly speeding up detection by incorporating a Region Proposal Network (RPN). Lin from Facebook [19] proposed the FPN algorithm, enhancing detection accuracy by fusing multi-scale feature maps and increasing information content. Cai et al. [20] proposed the Cascade R-CNN, which continuously optimizes detection results through cascading to improve detection accuracy. Duan et al. [21] introduced the CPN (Corner Proposal Network), which identifies areas of interest to extract candidate targets and classifies each, providing better capabilities to detect targets of various scales.

This study proposes a method for detecting dynamic targets underwater, which addresses issues such as inadequate underwater lighting, the presence of contaminants, and interference from moving objects. The algorithm enhances the performance of YOLOv8. This approach employs the YOLOv8 network as the fundamental framework of the algorithm, extends the feature extraction capability, and incorporates an attention mechanism to enhance the performance of eliminating dynamic interference and improving the accuracy of detection. The main contributions of this paper are as follows:

- An improved YOLOv8 algorithm for detecting targets in underwater scenes is put forward. This approach enhances the feature extraction layer of the YOLOv8 network and optimizes the convolutional network topology of Bottleneck, resulting in reduced computational requirements and improved detection accuracy.
- The enhanced YOLOv8 underwater scene target detection algorithm incorporates the enhanced SE attention mechanism to enhance the network’s feature extraction capabilities. Additionally, the network’s confidence box loss function is enhanced by replacing the CIoU loss function with the MPDIoU loss function. Significantly enhances the rate at which the model reaches convergence.
- The suggested underwater scene target identification algorithm underwent ablation experiments and comparison experiments. A dataset for detecting dynamic targets underwater was created, followed by conducting experiments. The experimental results confirmed the efficacy of the method.

The remainder of this paper is organized as follows: Section 2 proposes an improved YOLOv8 underwater dynamic target detection algorithm, including improving the convolutional network of the feature extraction layer, adding an improved SE attention mechanism, and replacing the CIoU loss function with the MPDIoU loss function. Section 3 explains the creation method of an underwater dynamic target detection data set and uses a self-built data set to conduct ablation experiments on the improved YOLOv8 network, and conducts experimental comparisons and evaluations with other target detection algorithms to verify the effectiveness of this algorithm. The advantages and disadvantages of the proposed method are then discussed in Section 4. Finally, Section 5 summarizes the research work performed and elaborates on the next work.

## 2. Improved YOLOv8 Underwater Dynamic Object Detection Method

### 2.1. YOLOv8-Based Object Detection Framework

**Figure 1:**
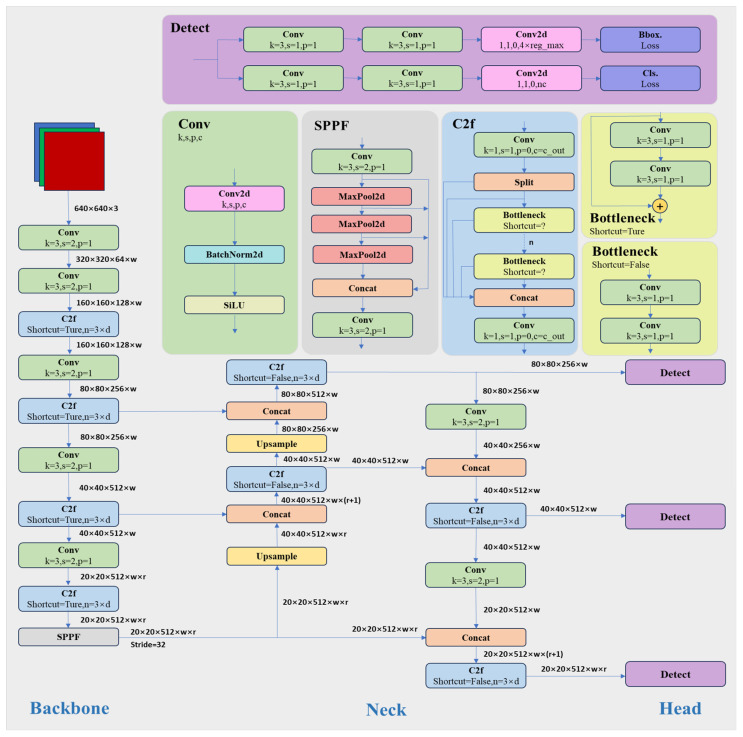
YOLOv8 network framework. The arrows in the diagram indicate sequential execution, where each module is performed one after the other upon completion of the previous module. In the upper-right corner of the diagram, the detailed components of each module are shown: the purple represents the detailed components of the Detect module, the light green represents the detailed components of the Conv module, the light gray represents the detailed components of the SPPF module, the blue represents the detailed components of the C2f module, and the yellow represents the detailed components of the Bottleneck module within the C2f module.

The comprehensive structure of the YOLOv8 algorithm is illustrated in Figure 1, comprising the Input, Backbone, Neck, and Head modules. The Input consists of an adaptive picture scaling module and a Mosaic data augmentation module. The Mosaic data augmentation module enhances data quality through techniques such as stitching and various other methods. A notable divergence from YOLOv5 is the integration of a method from YOLOX [22]. This method involves deactivating Mosaic throughout the last 10 epochs. Furthermore, adaptive picture scaling is utilized to scale photos to a specified dimension, hence significantly reducing the occurrence of black border padding. YOLOv8 is a model that operates independently of predefined anchor boxes. It directly forecasts the centers of objects, hence minimizing the number of box predictions and accelerating the Non-Maximum Suppression (NMS) procedure. NMS is a post-processing method employed to eliminate superfluous candidate detection boxes following inference.

The Backbone network, referred to as the feature extraction layer, is a deep network primarily employing the Darknet-53 architecture. This architecture comprises 52 convolutional layers in addition to an output layer. The system comprises three components: the Conv module, the CSP structure, and the SPPF module. This combination successfully addresses issues associated with gradient descent. The Conv module consists of convolution, BatchNorm, and SiLU activation functions. The CSP module was replaced by the C2f module to enhance light-weighting. The SPPF (SPP-Fast) module is an expedited spatial pyramid pooling model derived from SPP.

The Neck network architecture enhances the network’s ability to integrate features with the implementation of the PANnet structure [23]. This framework comprises a bidirectional route network that links a subordinate network to a superior network, facilitating the effective transmission of information from the lower tiers to the upper tier. The system utilizes upsampling and channel fusion techniques to enhance feature integration across many scales before inputting into the detection network. YOLOv8 has removed the convolutional structures in the upsampling phase and replaced the C3 module with the C2f module, unlike YOLOv5.

The Head layer has experienced significant alterations compared to YOLOv5. It has evolved from a coupled head architecture to a decoupled head architecture, like YOLOx (Decoupled-Head). This new configuration delineates the regression branch from the prediction branch. In the regression domain, the Distribution Focal Loss methodology employs the integral representation method to transform the prediction of regression coordinates from a deterministic singular value into a distribution. The YOLOv8 model incorporates the Task-Aligned Assigner approach, which effectively distributes positive and negative samples according to the TOOD framework. This technique dynamically modifies the allocation ratio of positive and negative samples during training and picks positive samples based on weighted scores from classification and regression. The procedure is depicted below.

(1) $$t=s^{\alpha }+u^{\beta }$$

where *s* represents the predicted score corresponding to the annotated category, and *u* is the IoU between the predicted box and the ground truth (GT) box. Multiplying these two factors measures the degree of Task-Alignment. *α* and *β* are weight hyperparameters, and *t* can control both the classification score and IoU optimization to achieve Task-Alignment, thereby guiding the network to dynamically focus on high-quality anchors. As the category score and IoU increase, the value of *t* approaches 1. The LOSS calculation includes two branches: classification and regression. For classification, the Sigmoid function is applied to estimate the likelihood for each category, using either VFL (Variance Focal Loss) or BCE (Binary Cross-Entropy) Loss as the classification loss function. The regression loss is divided into CIoU_Loss and Distribution Focal Loss components, where CIoU_Loss calculates the loss between the predicted box and the target box.

### 2.2. Improved YOLOv8 Underwater Dynamic Object Detection Method

#### 2.2.1. Bottleneck Convolutional Network Improvements

In the field of deep learning, numerous researchers have enhanced neural networks by minimizing the quantity of floating-point operations (FLOPs). Nevertheless, due to the inadequate performance of low floating-point operations per second, the reduction in FLOPs does not necessarily lead to a decrease in latency. In their study, Chen et al. [24] examined the operators involved in FLOPs and provided evidence that the low efficiency of floating-point operations is caused by frequent memory accesses. In response to this issue, they suggested the utilization of partial convolution (PConv) as a solution. PConv reduces the amount of floating point operations (FLOPs) and minimizes memory accesses, resulting in improved efficiency for feature extraction.

**Figure 2:**
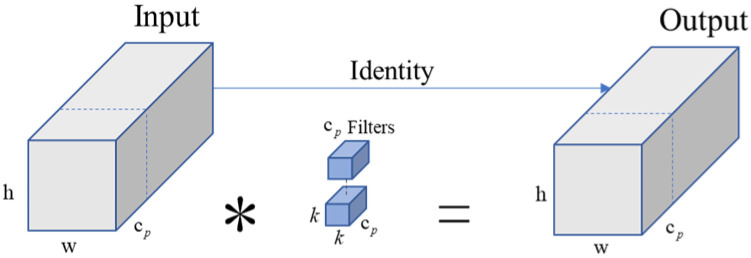
PConv (Partial Convolution) structure.

PConv not only reduces the redundancy of floating-point operations but also effectively reduces the number of memory accesses, showing impressive performance when replacing conventional convolutions in neural networks. Its network structure is illustrated in Figure 2. During spatial feature extraction, it performs standard convolution operations only on part of the channels, leaving the rest untouched, * represents convolution. When memory access is regular or sequential, either the first or the last continuous channels are treated as representative of the entire feature map for computation. It is assumed, without loss of generality, that the input and output feature maps have the same number of channels. Thus, the FLOP of PConv is only $h\times w\times k^{2}\times c_{p}^{2}$, The separation ratio formed by $c_{p}$ and c is $r=c_{p}/c$, when r=1/4 PConv only has 1/16 the FLOPS of Conv, it also has a smaller memory access volume, that is about $h\times w\times 2c_{p}$

**Figure 3:**
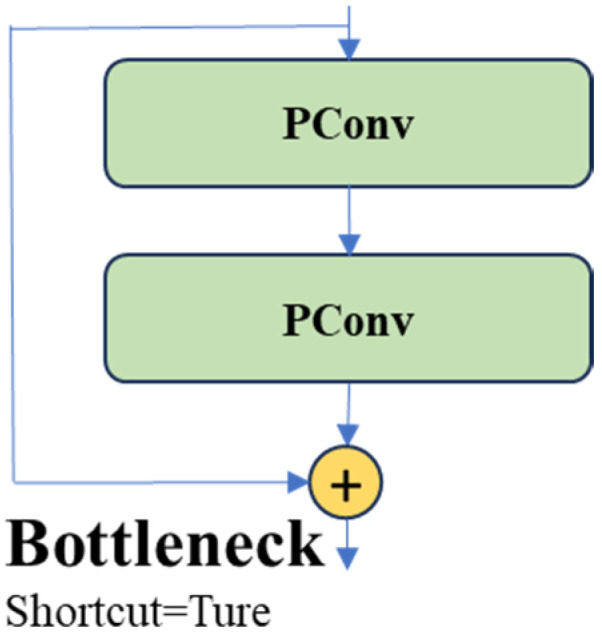
Improved Bottleneck structure.

This study primarily utilizes a PConv convolutional network to replace some convolutional structures in YOLOv8, addressing the problem of computational redundancy caused by the substantial similarity among different channel feature maps. This mostly entails substituting the Bottleneck convolutional network architecture, as depicted in Figure 3.

#### 2.2.2. Feature Extraction Layer Improvements

The main challenges in detecting dynamic objects underwater include the presence of small moving items, the similarity between dynamic objects and the background, and the significant decrease in light intensity. These characteristics often lead to errors in the detection of dynamic objects underwater, particularly in low-light imaging conditions affected by noise interference or when dynamic items blend with the background. In these circumstances, the feature extraction network may struggle to accurately capture dynamic object information, resulting in suboptimal training outcomes.

Attention mechanisms, inspired by biological visual systems, denote the capacity to focus on specific areas of interest when observing scenes. There are two categories of attention processes: spatial attention mechanisms and channel attention mechanisms. ECA-Net and SE-Net are classified as channel attention mechanisms, while CBAM, ResNet, SK-Net, and DANet integrate both channel and spatial attention mechanisms. This paper introduces the SE-Net attention mechanism [25] into the feature extraction layer to address issues in underwater dynamic object detection, replacing the activation function with PReLU (Parametric Rectified Linear Unit). This approach enhances the network’s capacity to extract properties of small dynamic underwater objects, thereby improving its ability to recognize and locate dynamic items in underwater environments.

**Figure 4:**
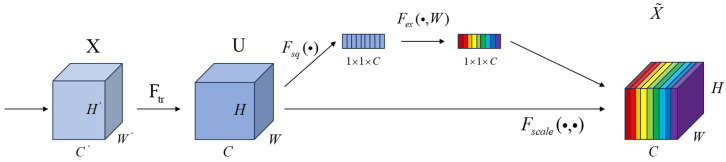
SE-net structure.

The main activities of the SE attention mechanism module are the squeeze and excitation processes, which evaluate the significance and interconnection of convolutional feature channels in order to enhance the network’s representation capabilities. The network structure is depicted in Figure 4. Feature map *X* undergoes operation $F_{tr}$ to produce feature map *U* is shown below.

(2) $$U_{c}=V_{c}*X=\sum_{s=1}^{c^{\prime }}*X^{s}$$

where $F_{tr}$ can be considered a standard convolution operator. In this formula, *V* represents a set of learned filter kernels, $V_{c}$ denotes the parameters of the *c*th filter. $V_{c}^{s}$ represents a 2D spatial kernel, ∗ signifies the convolution operation. Then, global information embedding is achieved through $F_{sq}$ as follows:

(3) $$Z_{c}=F_{sq}\left(U_{c}\right)=\frac{1}{H\times W}\sum_{i=1}^{H}\sum_{j=1}^{W}u\left(i,j\right)$$

where $Z_{c}$ is the *cth* element of *Z*. By using channel-wise global average pooling, the feature map with size *W* × *H* × *C* that contains global information is directly compressed into a feature vector with size 1 × 1 × *C*. This compresses the channel features of the *C* feature maps into a single value, enabling the generation of channel-level statistical data *Z* that contains contextual information and alleviates issues with channel dependencies. Then, adaptive recalibration (Excitation) is carried out using a gated mechanism composed of two fully connected layers. First, to reduce the computational cost, the first fully connected layer compresses the *C* channels into *C/r* channels, followed by a ReLU nonlinear activation layer. Next, the second fully connected layer restores the number of channels to *C* and uses a sigmoid activation function to obtain the weight s. This weight has dimensions 1 × 1 × *C* and is used to represent the weights of the *C* feature maps in the feature map *U*. Finally, a scaling operation is performed, applying the obtained attention weights to each channel’s features, which means each feature map in *U* is multiplied by the corresponding weight.

**Figure 5:**
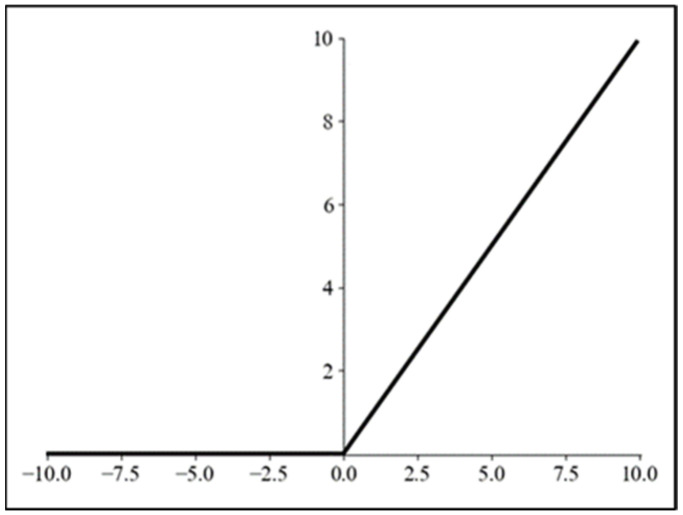
ReLU function curve.

In the $F_{ex}$ operation, the ReLU activation function is used. In the Excitation step, the first fully connected layer utilizes the ReLU activation function mapping [26], enhancing its nonlinear relationship with adjacent layers and thereby improving the performance in nonlinear tasks. The curve of the ReLU activation function is shown in Figure 5. As seen in the figure, when the input is less than or equal to zero, the output is set to zero. Since the slope of the function curve is zero, if there is a continuous presence of negative input, the output becomes meaningless, rendering the input node ineffective, which is what we commonly refer to as “dead neurons”.

**Figure 6:**
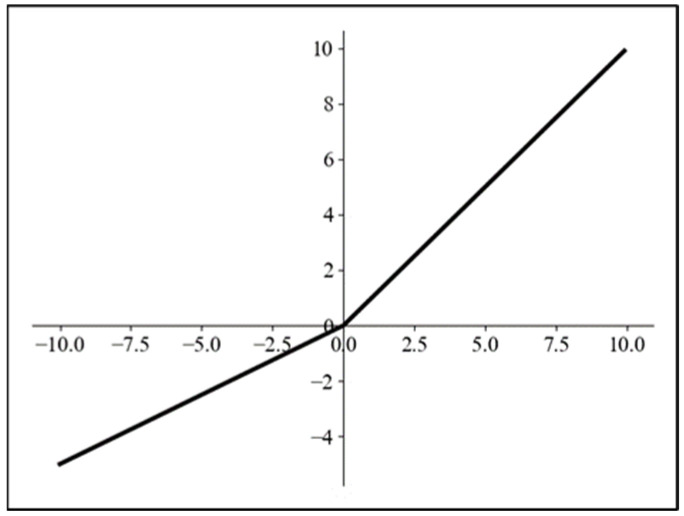
PReLU function curve.

To address the issue of ineffective input, nodes caused by zero output, Jing et al. [27] proposed the PReLU activation function based on the ReLU activation function. Its mathematical expression is shown below, and its function curve is shown in Figure 6.

(4) PRelux=axx    x<0x≥0

where *x* represents the input, and *a* represents an optimizable parameter, which determines the slope of the function curve in the negative axis.

#### 2.2.3. Loss Function Improvements

The loss function in object detection algorithms generally consists of three parts: classification loss, localization loss, and confidence loss. Classification loss measures the model’s accuracy in predicting the target class, while localization loss assesses the deviation between the model’s predicted bounding box and the ground truth bounding box. Confidence loss evaluates the model’s confidence in the detection results. In the YOLOv8 algorithm, the default localization loss function used is the CIoU [28] loss function, with its expression given by:

(5) $$CIoU=IoU-\frac{p^{2}\left(b,b^{gt}\right)}{c^{2}}-av$$

In object detection, Intersection over Union (IoU) is the ratio of the intersection area of the predicted bounding box and the ground truth bounding box to their union area. B represents the predicted bounding box, $b^{gt}$ is the ground truth bounding box, $p^{2}\left(b,b^{gt}\right)$ represents the Euclidean distance between the centers of the predicted and ground truth bounding boxes, *c* is the diagonal distance of the smallest rectangle that can encompass both the predicted and ground truth bounding boxes, and *α* is a coefficient related to IoU and is expressed as follows:

(6) $$\alpha =\frac{v}{1-IoU+v}$$

where *v* is a constant that plays a key role in the calculation process. Its calculation is as follows:

(7) $$v=\frac{4}{\pi^{2}}{\left(\arctan\frac{w^{gt}}{h^{gt}}-\arctan\frac{w}{h}\right)}^{2}$$

where $w^{gt}$ and $h^{gt}$ represent the width and height of the actual ground truth bounding box, respectively; *w*, *h* represents the width and height of the predicted bounding box.

CIoU (Complete Intersection over Union) loss can be ineffective for optimization when the predicted and ground truth bounding boxes have the same aspect ratio, but their widths and heights are significantly different. Additionally, the calculation process can be complex. Therefore, in this work, the CIoU loss function in YOLOv8 is replaced with the MPDIoU (Maximal Probability Distribution Intersection over Union) loss function [29]. The MPDIoU loss function is expressed in Equation (8).

(8) $$MPDIoU=IoU-\frac{d_{1}^{2}}{w^{2}+h^{2}}-\frac{d_{2}^{2}}{w^{2}+h^{2}}$$

where,

(9) $$\left\{\begin{array}{c} d_{1}^{2}={\left(x_{1}^{prd}-x_{1}^{gt}\right)}^{2}+{\left(y_{1}^{prd}-y_{1}^{gt}\right)}^{2}\\ d_{2}^{2}={\left(x_{2}^{prd}-x_{2}^{gt}\right)}^{2}+{\left(y_{2}^{prd}-y_{2}^{gt}\right)}^{2} \end{array}\right.$$

**Figure 7:**
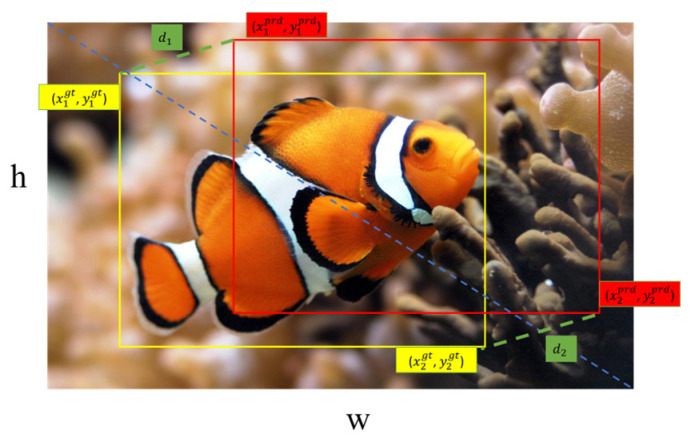
MPDIoU parameter diagram.

The specific parameters are shown in Figure 7.

## 3. Experimental Results and Analysis

### 3.1. Experimental Setup

The underwater dynamic object detection method for ORBSLAM3 primarily depends on the augmented YOLOv8 algorithm for underwater object detection. The enhanced YOLOv8 network is trained on a Dell desktop PC. The training outcomes are subsequently analyzed, and the trained model is employed to identify and conduct experimental investigations on novel images. Table 1 presents a summary of the software and hardware configuration employed in this study.

### 3.2. Dataset Creation

To improve the precision of recognizing moving objects underwater through deep learning algorithms, a substantial dataset tailored for underwater dynamic targets is essential. This dataset must include a significant volume of data, representing diverse scenarios, addressing all standard dynamic target categories, and providing accurate annotations. Consequently, based on these parameters, our work created a dataset explicitly tailored for dynamic target detection.

This work utilizes a manual method to distinguish between static and dynamic items to create a dataset, as conventional object detection algorithms struggle to accurately identify dynamic targets. Entities capable of movement, such as humans, fish, turtles, and jellyfish, are classified as dynamic and designated as dynamically labeled. Conversely, entities such as boulders, coral, and aquatic vegetation are considered fixed and do not receive any sort of designation. This technique facilitates the identification and removal of moving objects during the creation of a three-dimensional representation, so effectively mitigating disturbances generated by such items in underwater scene reconstructions.

**Figure 8:**
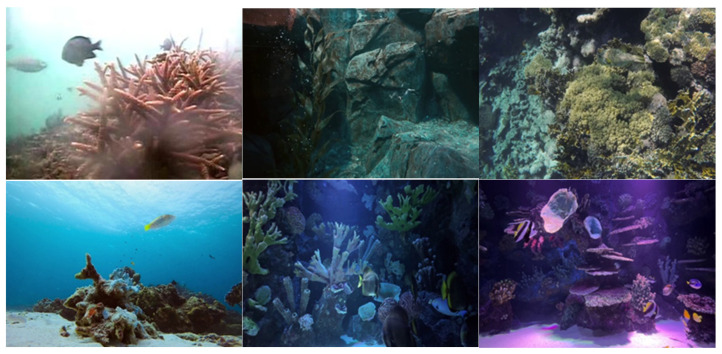
Dynamic target detection dataset.

To achieve more precise identification outcomes of underwater dynamic objects utilizing deep learning, it is essential to endure extensive training with a substantial dataset. The dataset employed in this study mostly consists of data sourced from the high-definition video platform Pexel, supplemented by a segment derived from the RUOD dataset. The scenarios display a varied array of characteristics, including small dynamic targets, dynamic targets that blend with the background, and scenarios featuring many dynamic targets. The dynamic target categories include a variety of aquatic organisms, such as various fish species, jellyfish, turtles, and scuba divers. The labeling process was conducted via LabelImg software, explicitly designating the label “dynamic” to the recognized items. Figure 8 illustrates a segment of the image data.

### 3.3. Experimental Results and Analysis

The approach shown in this chapter for identifying moving objects in underwater environments and reconstructing a 3D representation of the scene relies on an enhanced YOLOv8 neural network. The training of the enhanced YOLOv8 network predominantly utilizes a self-constructed dynamic image dataset labeled as “dynamic”. The dimensions of the input image are specified as 640 × 640 × 3, and the starting learning rate is set to 0.002. The batch size is configured as 16, whereas the number of epochs is specified as 100. The model utilized is yolov8n.pt. The model that had undergone training was subsequently employed to identify additional photos, and the outcomes of both the training and identification processes were stored in the designated run folder.

The experiment design mainly consists of ablation experiments and comparison tests. The former objective is to assess the influence of various enhancements on the outcomes of model training, whilst the latter objective is to validate the efficacy of the algorithm provided in this study. In addition, the training results were assessed both before and after the improvements were made.

#### 3.3.1. Ablation Experiments

The ablation experiment entails the comparison of various experimental models within regulated experimental settings and beginning parameters. Ablation experiments can clearly highlight the influence of each improvement on the experimental findings, facilitating a direct comparison of the algorithm’s advantages. In the paper, yolov8n represents training using the pretrained yolov8n model. yolov8n+CBAM, yolov8n+ECA, and yolov8n+SE represent training with the addition of CBAM, ECA, and SE attention mechanisms, respectively. yolov8n+Pconv refers to training with modified convolutional networks. yolov8n+Pconv+SE represents training on the basis of the modified convolutional networks with the addition of the attention mechanism network. yolov8n+Pconv+P-SE refers to training with modified convolutional networks and the addition of improved attention mechanism networks. yolov8n+Pconv+P-SE+MPDIoU refers to training with modified convolutional networks, the addition of improved attention mechanism networks, and improved loss function. Table 2 displays the outcomes of the ablation experiment.

According to the table, the SE attention mechanism demonstrates superior performance compared to the other two regularly utilized attention mechanisms on this dataset. When comparing the yolov8n+Pconv model with the original yolov8n model, there was a 0.6% increase in average precision and a reduction of 0.89M in parameter size. This suggests that replacing certain parts of the original convolutional network with the Pconv network resulted in a smaller parameter size and improved accuracy of the model. When comparing yolov8n+Pconv+SE with yolov8n+Pconv, the average precision increased by 0.2%. This suggests that the SE attention mechanism improves the network‘s capability to extract important features. When comparing yolov8n+Pconv+P-SE with yolov8n+Pconv+SE, the average precision showed a 0.4% gain. This indicates that altering the SE net activation function resulted in higher accuracy for the model. When comparing yolov8n+Pconv+P-SE+MPDIoU with yolov8n+Pconv+P-SE, the average precision increased by 0.4%. This suggests that the addition of the MPDIoU loss function effectively resolved the limitations of the CIoU loss function, resulting in a considerable enhancement in the model‘s convergence speed and accuracy. The algorithm‘s overall average precision significantly improved compared to the original yolov8n network model, showcasing the efficacy of the suggested technique.

#### 3.3.2. Analysis of the Training Process

**Figure 9:**
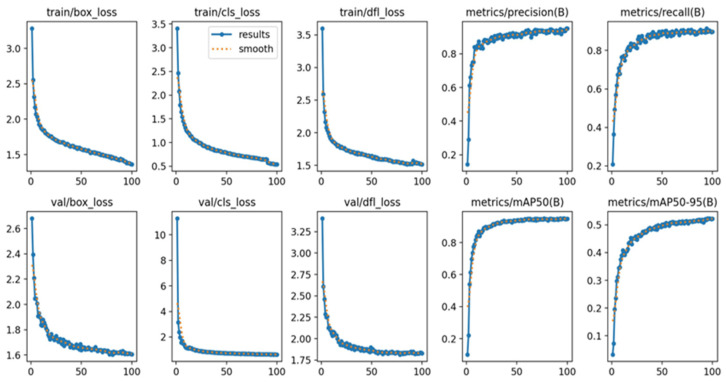
Original YOLOv8 network training result graph.

**Figure 10:**
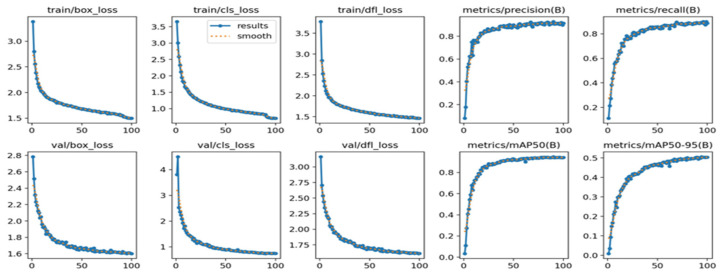
Improved YOLOv8 network training result graph.

In order to gain a deeper comprehension of the model‘s superiority during the training process, we carefully examined and studied the training outcomes both prior to and following the enhancements. The following diagrams depict the outcomes: Figure 9 displays the training outcomes of the original YOLOv8 network, whereas Figure 10 showcases the training outcomes of the enhanced YOLOv8 network.

Analysis of the data reveals that the precision and recall curves of the improved YOLOv8 network demonstrate a more consistent and stable trajectory, with diminished oscillations.

#### 3.3.3. Comparative Experiments of Different Models

To evaluate the effectiveness of the method outlined in this research, it was trained on a dataset that was specifically developed for this purpose. The algorithm was subsequently evaluated against commonly utilized object detection algorithms. An analysis was performed on the performance of each method, with the experimental findings presented in Table 3.

Table 3 illustrates that the suggested method surpasses the Faster RCNN two-stage object detection algorithm regarding mean average precision (mAP), despite the latter exhibiting comparatively high detection accuracy. Moreover, in comparison to prior single-stage object detection algorithms, the proposed method demonstrates enhanced average precision while maintaining a competitive inference time. This illustrates the superiority of the upgraded network on the self-assembled dynamic object identification dataset.

#### 3.3.4. Detection Results and Analysis

**Figure 11:**
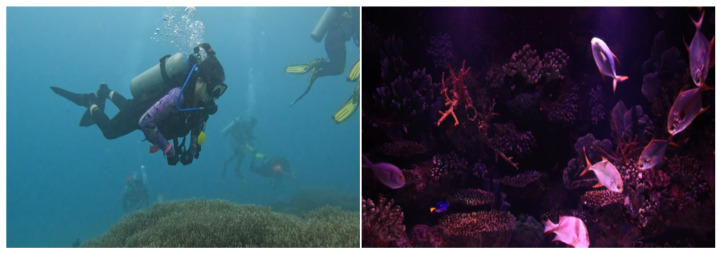
The original images.

**Figure 12:**
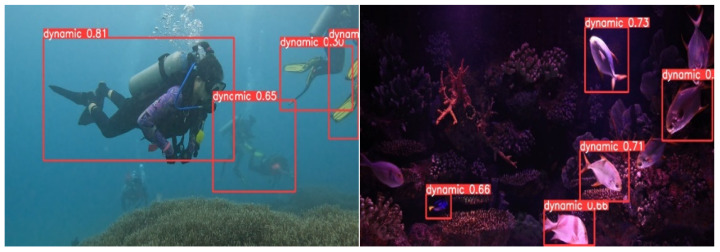
The detection results using the unmodified YOLOv8 algorithm.

**Figure 13:**
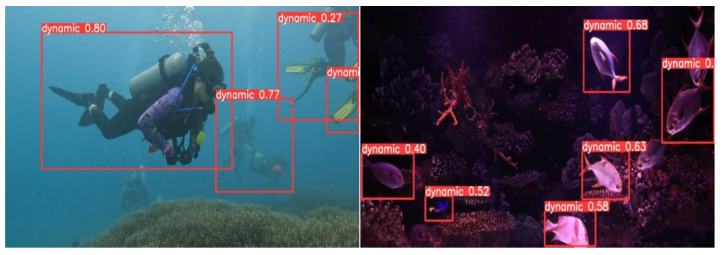
The detection results using the proposed modified YOLOv8 algorithm.

To evaluate the subjective performance of the results derived from the proposed algorithm, a selection of representative images was made. The photographs encompassed those captured in low-light environments, those in which the targets were camouflaged with the background, and those where the targets were partially obscured. The original and revised YOLOv8 algorithms were employed for picture detection, facilitating comparison and analysis. Figure 11, Figure 12 and Figure 13 illustrate the original images, the detection results achieved with the standard YOLOv8 approach, and the detection results acquired with the suggested modified YOLOv8 algorithm, respectively.

Analysis of the images reveals that the enhanced method exhibits superior precision in accurately delineating the diver in the first image, which presents occlusion issues, in contrast to the original YOLOv8 technique. In the second image, where the target closely matches the background and certain areas are poorly illuminated, the advanced algorithm demonstrates a superior capacity to detect targets in low-light conditions, hence indicating enhanced performance.

## 4. Discussion

Accurate detection is essential to eliminate dynamic interference, which affects the reconstruction of underwater 3D environments. This work utilizes a method of manually distinguishing between dynamic and static items to create a dataset for the detection of dynamic entities underwater. Furthermore, it introduces an improved iteration of the YOLOv8 algorithm for the detection of moving objects in aquatic settings. The efficacy of each modification is illustrated by ablation experiments, while comparisons with current object detection algorithms substantiate the effectiveness of the proposed method on the custom-built dataset.

The technique of constructing a dataset through the human distinction of moving and stationary items, along with an advanced YOLOv8 algorithm for detecting moving objects underwater, can be efficiently utilized to recognize dynamic disturbances in underwater 3D scene reconstruction. Nevertheless, the dataset created by the authors is somewhat tiny, and manually curated datasets are frequently limited in quantity, potentially resulting in models that overfit in particular contexts and exhibit subpar performance in novel surroundings. Furthermore, hand annotation is readily influenced by subjective considerations. Diverse annotators may categorize the identical object variably, leading to conflicting labels within the dataset. The human annotation procedure is labor-intensive and expensive, particularly for large-scale datasets, potentially constraining their growth and diversity. Consequently, enhancing the dataset may be a valuable later action.

## 5. Conclusions

Underwater 3D scene reconstruction frequently encounters dynamic interference, making it crucial to precisely detect and effectively eliminate such interference throughout the reconstruction process. This study utilizes the advanced YOLOv8 algorithm as a basis and presents an enhanced underwater dynamic object identification system, considering the challenging underwater conditions and limited daylight. Furthermore, it presents a self-constructed dataset for detecting dynamic interference in underwater environments. The experimental findings demonstrate that the enhanced YOLOv8 algorithm attains a mean average precision (mAP) of 95.1%, hence facilitating more precise identification of moving objects in underwater environments, particularly small items in intricate underwater settings.

Our ongoing work will be directed towards enhancing the self-constructed dataset for detecting underwater dynamic objects. This will involve improving the diversity of scenarios and broadening the range of categories for underwater dynamic objects. Furthermore, it is crucial to continuously optimize the object detection algorithm in order to attain ever more accurate detection outcomes.

## Figures and Tables

**Table 1:** Experimental platform and environment configuration.

Software Environment	PyCharm2020 + Anaconda3 + Python3.8 + Pytorch1.18.0	
hardware environment	Dell desktop computer	operating system: Windows11
		processor: AMD Ryzen 7 5800H
		graphics card: NVIDIA GeForce RTX 4060

**Table 2:** Ablation experiment results.

Network	mAP@0.5 (%)	Param/M	Time (ms)
yolov8n	93.5	3.01	1.0
yolov8n+CBAM	93.8	3.08	1.9
yolov8n+ECA	93.5	3.01	1.2
yolov8n+SE	94.0	3.01	1.3
yolov8n+Pconv	94.1	2.12	2.5
yolov8n+Pconv+SE	94.3	2.12	1.5
yolov8n+Pconv+P-SE	94.7	2.12	1.5
yolov8n+Pconv+P-SE+MPDIoU	**95.1**	**2.12**	**2.2**

**Table 3:** Algorithm Comparison.

Evaluation Criteria	Proposed Algorithm	YOLOv5	SSD	YOLOv4	Faster RCNN
mAP@0.5 (%)	**95.1**	91	89	85	87
Time (ms)	**2.2**	2.1	2.3	2.8	2.5

## Data Availability

The datasets used or analysed during the current study are available from the corresponding author upon reasonable request.
